# Preparation of Microcapsules Using a Poly(2-(dimethylamino)ethyl methacrylate)‑*b*‑poly(benzyl methacrylate) Diblock Copolymer Emulsifier

**DOI:** 10.1021/acs.langmuir.5c04262

**Published:** 2025-12-24

**Authors:** Viktor Kallebäck, Csilla György, Gustav Eriksson, Steven P. Armes, Markus L. Andersson Trojer, Lars Evenäs

**Affiliations:** † Department of Sustainable Material Systems, 388792 RISE Research Institutes of Sweden, 431 53 Mölndal, Sweden; ‡ Department of Chemistry and Chemical Engineering, 211731 Chalmers University of Technology, 412 96 Gothenburg, Sweden; § Dainton Building, School of Mathematical and Physical Sciences, 7315 University of Sheffield, Sheffield, Brook Hill, Sheffield, South Yorkshire S3 7HF, U.K.

## Abstract

Most encapsulation techniques require suitable polymers or surfactants to stabilize the microcapsule suspension. Typically, such stabilizers are present in excess in the aqueous continuous phase. In this study, the use of a poly­(2-(dimethylamino)­ethyl methacrylate)-poly­(benzyl methacrylate) (PDMA–PBzMA) diblock copolymer is examined as an efficient stabilizer for the preparation of three types of methacrylic microcapsules with different morphologies. Unlike conventional water-soluble stabilizers, this amphiphilic copolymer is dissolved directly in the dispersed organic phase. This approach minimizes the amount of stabilizer present in the aqueous continuous phase and hence maximizes the formulation efficiency. This new PDMA–PBzMA stabilizer enables either poly­(benzyl methacrylate) (PBzMA) or poly­(methyl methacrylate) (PMMA) microcapsules to be prepared with excellent colloidal stability and shell integrity, comparable to that previously achieved using poly­(vinyl alcohol). Such formulations can produce (*i*) monolithic microcapsules, (*ii*) oil-core microcapsules via internal phase separation, or (*iii*) aqueous-core microcapsules via water-in-oil-in-water (*w*_1_/*o*/*w*_2_) double emulsification. In particular, PBzMA microcapsules exhibited an exceptionally slow release of the encapsulated hydrophobic model active pyrene. Diffusivities of the encapsulated active species on the order of 1 × 10^–20^ m^2^ s^–1^ were determined by fitting release data to appropriate models assuming Fickian diffusion. For such microcapsules, the PBzMA shell matrix acted as the primary release-rate-limiting factor. Given that PDMA–PBzMA diblock copolymers can be conveniently prepared via polymerization-induced self-assembly (PISA), this study highlights their potential as a versatile, efficient stabilizer for a broad range of microcapsule formulations.

## Graphical abstract

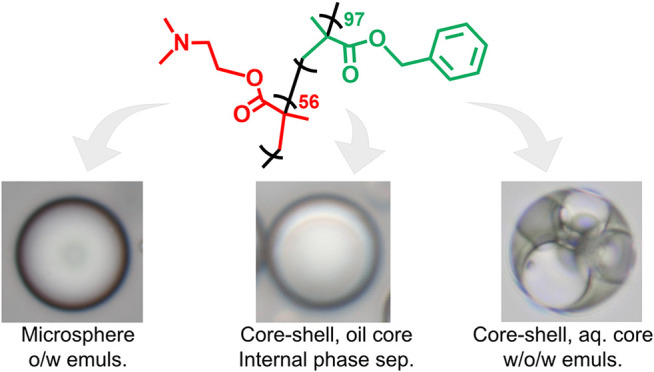

## Introduction

Microcapsules are utilized for a broad range of applications, ranging from antifouling and self-healing coatings to pharmaceuticals and food additives.^1–6^ The benefits of encapsulating active substances are twofold. First, the microcapsule protects the active species from environmental degradation. Second, the release rate of active substances can be controlled, thus enabling precise and adjustable delivery over time. Release profiles can vary from relatively slow sustained^4^ release over extended time periods (hours/days/weeks) to rapid triggered^7^ release in response to external stimuli such as temperature, pH, electrolyte concentration, redox chemistry, or UV irradiation.

The microcapsule morphology is critical for optimal performance in terms of both protection and controlled release. A detailed study^8^ on the thermodynamic and kinetic factors controlling microcapsule formulation following the internal phase separation pathway, first described by Loxley and Vincent,^9^ has recently been published. Such formulations involve an aqueous emulsion comprising droplets of an organic solvent that contain all of the components required to produce the microcapsules, which are subsequently formed through internal phase separation by solvent evaporation at controlled conditions. Interfacial tensions, and by extension the type of stabilizer used during formulation, play a key role in determining the final microcapsule morphology. Poly­(vinyl alcohol) (PVA) is the most widely used stabilizer for such microcapsule formulations.^9–11^ It provides the spreading coefficients^8,9^ that are required to ensure encapsulation and produces sterically stabilized microcapsules, thus minimizing their aggregation.

However, the PVA concentration used for microcapsule formulation is relatively high (typically 1% w/w in the continuous aqueous phase). Moreover, less than one tenth of this PVA adsorbs at the surface of the microcapsules, with the majority remaining in the continuous aqueous phase.^12^ Depending on the intended application, additional postformulation steps (e.g., filtration or centrifugation) may be required to remove this excess PVA. This limits the resource efficiency as well as the scalability and practicality of certain microcapsule applications. A more efficient approach would involve stabilizing microcapsules from within the dispersed organic droplets, thus eliminating excess stabilizer in the aqueous phase and minimizing the need for microcapsule purification.

Previously, certain amphiphilic diblock copolymers, such as poly­(sodium methacrylate)-poly­(methyl methacrylate), have been identified as effective dispersants for core–shell microcapsules.^8,13^ Such copolymers combine a hydrophilic poly­(sodium methacrylate) block that strongly interacts with water and a hydrophobic poly­(methyl methacrylate) block that is compatible with the microcapsule shells. However, optimal performance requires a relatively short hydrophobic block and a relatively long hydrophilic block. Unfortunately, such diblock dispersants must be dissolved in the aqueous continuous phase rather than the organic droplet phase. Lower copolymer concentrations are required compared to conventional stabilizers such as PVA but excess nonadsorbed copolymer nevertheless remains in the aqueous continuous phase after microcapsule formation.

It is well-known that reversible addition–fragmentation chain transfer (RAFT) polymerization enables the convenient synthesis of a wide range of functional vinyl polymers.^14–16^ Herein, an amphiphilic poly­(2-(dimethylamino)­ethyl methacrylate)-poly­(benzyl methacrylate) (PDMA–PBzMA) diblock copolymer has been prepared via RAFT-mediated polymerization-induced self-assembly (PISA).^17,18^ This study focuses on demonstrating the feasibility and versatility of PDMA–PBzMA as a stabilizer across multiple microcapsule morphologies. Given its PBzMA-rich composition, this copolymer acts as a water-insoluble polymeric emulsifier/stabilizer for microcapsule formation. This approach is much more efficient in terms of the required stabilizer concentration, and little or no stabilizer migrates to the aqueous continuous phase during microcapsule formation, which aids purification. Moreover, several microcapsule morphologies can be accessed using this new emulsifier/stabilizer.

## Materials and Methods

2-(Dimethylamino)­ethyl methacrylate (DMA, 98%), benzyl methacrylate (BzMA, 96%), 2-cyano-2-propyl benzodithioate (CPDB, > 97%), poly­(benzyl methacrylate) (PBzMA, 100 kg mol^–1^), dichloromethane (DCM, ≥99.8%), perylene (≥99%), pyrene (98%), 9,10-bis­[(triisopropylsilyl)­ethynyl]-anthracene (TIPS-An, >99%), hexadecane (HD, 99%), sodium phosphate (monobasic and dibasic, 99%), tris­(hydroxymethyl)­aminomethane (TRIS, ≥99.8%), sodium chloride (99%), Brij L23, and deuterated chloroform (CDCl_3_, 99.8%) were purchased from Merck and used as received. 2,2′-Azobis­(isobutyronitrile) (AIBN) was purchased from Molekula while poly­(vinyl alcohol) (PVA, 95% hydrolyzed, 95 kg mol^–1^) was obtained from Acros Organics. Poly­(methyl methacrylate) (PMMA, 25 kg mol^–1^) was purchased from Polysciences. Bermocoll E230 was a gift from Nouryon. Tetrahydrofuran, ethanol, hydrochloric acid, and petroleum ether were obtained from VWR Chemicals. Water of Milli-Q purity (18.2 MΩ·cm, Millipore) was used throughout the work.

### Synthesis of Poly­(2-(dimethylamino)­ethyl methacrylate) (PDMA_56_) Precursor via RAFT Solution Polymerization of DMA in Tetrahydrofuran

DMA monomer (17.05 g, 0.11 mol; target DP = 60), CPDB (0.4 g, 1.81 mmol), and AIBN initiator (59.35 mg, 0.36 mmol, CPDB/AIBN molar ratio = 5.0) were dissolved in tetrahydrofuran (11.67 g) to afford a 60% w/w solution in a sealed round-bottomed flask containing a magnetic stir bar. This flask was deoxygenated with a stream of N_2_ gas for 30 min, and the degassed reaction mixture was then heated to 70 °C with magnetic stirring. After 17 h, the DMA polymerization was quenched by exposing the reaction mixture to air while cooling the flask to 20 °C. A final DMA conversion of 90% was determined by comparing the integrated oxymethylene signals of the monomer at 4.21 and 4.26 ppm with the oxymethylene signals assigned to the polymerized DMA units at 3.95 and 4.15 ppm using ^1^H NMR spectroscopy. The crude PDMA_56_ was precipitated twice into a 10-fold excess of petroleum ether. Then, the purified precursor was dried under a vacuum overnight to produce a pink solid. The mean DP was determined to be 56 via ^1^H NMR spectroscopy by comparing the five aromatic phenyl protons assigned to the dithiobenzoate end-group at 7.30–7.90 ppm with the oxymethylene protons assigned to the polymerized DMA units at 3.95–4.15 ppm (Figure S1). Chloroform GPC studies indicated a *M*_*n*_ of 6.2 kg mol^–1^ and a *M*_w_/*M*_n_ of 1.19 (Figure S2).

### Synthesis of Poly­(2-(dimethylamino)­ethyl methacrylate)-poly­(benzyl methacrylate) (PDMA_56_-PBzMA_97_) Diblock Copolymer via RAFT Alcoholic Dispersion Polymerization of BzMA in Ethanol

PDMA_56_ precursor (14.41 g; 1.60 mmol), AIBN initiator (52.44 mg; 0.32 mmol, precursor/AIBN molar ratio = 5.0), BzMA monomer (28.13 g; 0.16 mol; target DP = 100) and ethanol (216 mL) were weighed into a round-bottom flask, which was immersed in an ice bath and purged with nitrogen for 30 min. Then the sealed vial was immersed in a preheated oil bath at 70 °C and the reaction mixture was magnetically stirred for 20 h. ^1^H NMR analysis indicated 97% BzMA conversion by comparing the integrated vinyl signal of the BzMA monomer at 6.09−6.12 ppm to the integrated aromatic signal of the PBzMA at 7.10–7.35 ppm. An assigned ^1^H NMR spectrum was recorded in CD_2_Cl_2_ for the purified diblock copolymer (see Figure S1). The integral for signal *a* at ∼4.1 ppm assigned to the two oxymethylene protons next to the methacrylic ester for the 2-(dimethylamino)­ethyl methacrylate repeat units was compared to that for signal *d* at ∼4.9 ppm, which is assigned to the two benzylic protons for the benzyl methacrylate repeat units. Thus, the diblock copolymer composition was calculated to be PDMA_56_-PBzMA_97_. Chloroform GPC analysis indicated an *M*_n_ of 20.2 kg mol^–1^ and an *M*_w_/*M*_n_ of 1.18 (Figure S2). The PDMA_56_-PBzMA_97_ diblock copolymer was purified by three consecutive precipitations into a 10-fold excess of petroleum ether (with redissolution in THF) followed by filtration and drying under vacuum.

#### Microcapsule Formulation

Monolithic microcapsules and oil core-polymer shell microcapsules were prepared following the internal phase separation by the solvent evaporation method, first reported by Vincent and Loxley^9^ and subsequently modified by Eriksson et al.^8^ Aqueous-core microcapsules were prepared following a double emulsification (*w*_1_/*o*/*w*_2_) route.^19^

##### Monolithic Microcapsules and Oil Core-Polymer Shell Microcapsules

An organic phase was prepared where all the microcapsule components (e.g., PBzMA or PMMA shell polymer, active substance (pyrene, perylene, or TIPS-An), hexadecane core oil (for core–shell particles), and PDMA_56_-PBzMA_97_ stabilizer) at a total mass of 100 mg were dissolved in 2.4 mL of DCM. For the monolithic microcapsules, pyrene or perylene was used as a model active and added at 1% w/w based on the total microcapsule mass. Here, perylene was used for fluorescence microscopy studies due to its favorable matching of excitation and emission wavelengths with the microscope filter sets, while pyrene was used in the release measurements. For the core–shell microcapsules, a shell-to-core mass ratio (*m*_s_/*m*_c_) of 3.0 was used, and 0.6% w/w TIPS-An (based on the core oil mass) was encapsulated. TIPS-An was used for its high solubility in the core oil and its favorable partitioning toward this phase. The PDMA_56_-PBzMA_97_ stabilizer was added at 2% (w/w) based on the shell polymer for either the monolithic microcapsules or the core–shell microcapsules. The prepared organic phase (2.4 mL) was added slowly to the aqueous phase (2.5 mL) containing 2 mM HCl while stirring at 4000 rpm. A 1% (w/w) aqueous PVA solution was used as an alternative to the PDMA_56_-PBzMA_97_ stabilizer for the preparation of reference formulations. Emulsification was conducted for 60 min at ambient conditions using a Kinematica Polytron PT3100D drive unit equipped with dispersing aggregate PT-DA 07/2EC-E107. After homogenization, the emulsion was diluted with water (5.0 mL) and magnetically stirred at 300 rpm overnight at ambient conditions to ensure complete evaporation of the volatile DCM solvent.

##### Aqueous Core-Polymer Shell Microcapsules

An organic phase was prepared by dissolving 100 mg of PBzMA and 2 mg of PDMA–PBzMA in DCM (1.0 mL). Under shearing at 15,000 rpm, an aqueous phase containing 2% w/w Bermocoll E230 at pH 4 as a rheology modifier was added. Emulsification was carried out for 5 min under ambient conditions using a Kinematica Polytron PT3100D drive unit equipped with dispersing aggregate PT-DA 07/2EC-E107. The formed w/o emulsion was then emulsified into an aqueous phase (3.0 mL) containing 2 mM HCl at a shearing speed of 2500 rpm for 5 min. Finally, the formed *w*_1_/*o*/*w*_2_ emulsion was diluted with water (5.0 mL) and magnetically stirred at 300 rpm overnight at ambient conditions to ensure complete evaporation of the volatile DCM solvent.

#### Release Studies

##### Measuring the Fractional Release of Encapsulated Actives

The principle for determining the rate of release of actives from microcapsules has been previously described in detail by us.^20^ Briefly, a known amount of microcapsule suspension was added to a larger volume of the selected release medium containing 6% (w/w) Brij L23. Release measurements were carried out under both acidic and weakly basic conditions. The acidic release medium (adjusted to pH 3) was buffered with 10 mM phosphate buffer, while the weakly basic release medium (adjusted to pH 9) was buffered with 10 mM TRIS. In each case, the microcapsule suspension was magnetically stirred at 37.0 ± 0.1 °C in an incubator.

At predetermined time intervals, aliquots (1.5 mL) were extracted from the release medium and centrifuged (17,000 × *g*, 5 min). The supernatant was extracted and analyzed using a HP 8453 UV–visible spectrophotometer to determine the concentration of released pyrene. The total amount of pyrene (*m*_tot_) was determined by adding a 1.0 mL aliquot of the microcapsule-containing release medium to ethanol (3.0 mL), placing this sample on a reciprocating shaker overnight, and finally separating the microcapsules from the aqueous continuous phase via centrifugation prior to determining the concentration of pyrene spectrophotometrically as described above. This quantification was based on the Beer–Lambert law with a determined molar extinction coefficient for pyrene of 56,630 M^–1^ cm^–1^ at 242 nm.^21^

##### Release Models

A Fickian diffusion model was used to describe the experimental release data, where analytical solutions have been derived by Crank^22^ and previously used by us to describe and model microcapsule release data.^20^ The fractional release from a sphere, *f*_s_, at time *t* is described by

1 $$f_{s}\left(D,r,t\right)=\frac{\alpha_{s}}{1+\alpha_{s}}\left(1-\sum_{n=1}^{\infty }\frac{6\alpha_{s}\left(\alpha_{s}+1\right)}{9+9\alpha_{s}+q_{s,n}^{2}\alpha_{s}^{2}}\exp\left(-\frac{Dq_{s,n}^{2}t}{r^{2}}\right)\right)$$

where *D* is the diffusion coefficient of the encapsulated active substance in a sphere with radius *r*. The coefficient α_s_ is defined as

2 $$\alpha_{s}=\frac{V_{\mathrm{sink}}}{V_{\mathrm{sphere}}K}$$

Here, *K* is the partition coefficient of the active substance between the release medium and sphere, and *V*_sink_ and *V*_sphere_ are their respective total volumes. Finally, *q*_s,*n*_ is the *n:*th nonzero positive root of

3 $$\tan q_{s,n}=\frac{3q_{s,n}}{3+\alpha_{s}q_{s,n}^{2}}$$

The microcapsules are polydisperse with a characteristic size distribution *p*(*r*). The final expression for the fractional release from polydisperse microcapsules is then given by

4 $$f_{s,\mathrm{pd}}\left(D,t\right)=\frac{m\left(t\right)}{m_{\mathrm{tot}}}=\frac{\int f_{s}\left(r,t\right)p\left(r\right)r^{3}dr}{\int p\left(r\right)r^{3}dr}$$

where *m* is the mass of released active substance and *m*_tot_ is the total amount in the sphere at time zero. As shown in Figure S3, the microcapsule radii were well described by a log-normal size distribution

5 $$p\left(r\right)=\frac{1}{r\sigma \sqrt{2\pi }}\exp\left(-\frac{{\left(\ln x-\mu \right)}^{2}}{2\sigma^{2}}\right)$$

where μ and σ are the mean and standard deviation of the logarithmized radius.

#### Characterization Methods

##### ^1^H NMR Spectroscopy

^1^H NMR spectra were recorded in CDCl_3_ by using a 400 MHz Bruker Avance spectrometer. Typically, 64 scans were averaged per spectrum.

##### Gel Permeation Chromatography (GPC)

GPC analysis was conducted at 35 °C by using a chloroform eluent containing 2 mM LiBr at a flow rate of 1.0 mL min^–1^. The instrument setup comprised an Agilent 1260 GPC system, two Agilent PL gel 5 mm Mixed-C columns connected in series with a guard column and a refractive index detector. Calibration was achieved using a series of eight near-monodisperse poly­(methyl methacrylate) (PMMA) standards with *M*_*p*_ values ranging from 800 to 988,000 g mol^–1^.

##### Optical Microscopy

Micrographs were acquired using a Zeiss Axio Imager Z2m equipped with a Zeiss Colibri 7 illumination source and filter set 49 for fluorescence imaging. A combination of brightfield, differential interference contrast, and fluorescence was employed for analysis.

##### Electrophoretic Light Scattering

ζ-Potentials were measured using an Anton Paar Litesizer 500 instrument at 25 °C. The as-formulated microcapsule suspensions were diluted to 0.01% w/w in 1 mM NaCl and placed in Omega cuvettes prior to measurement. A colloidal dispersion of PDMA–PBzMA nanoparticles was prepared by dispersing PDMA_56_-PBzMA_97_ dry powder (100 mg) in 1 mM NaCl (7.5 mL) for 10 min with the aid of a VWR ultrasonic bath.

## Results and Discussion

### Synthesis of PDMA–PBzMA Diblock Copolymer

The synthesis of PDMA_56_-PBzMA_97_ in the form of sterically stabilized diblock copolymer nanoparticles was conducted in two steps. First, a poly­(2-(dimethylamino)­ethyl methacrylate) (PDMA) precursor was prepared via RAFT solution polymerization. Then this precursor was chain-extended via RAFT alcoholic dispersion polymerization of benzyl methacrylate (BzMA).^17,18^ This PISA synthesis is outlined in Scheme 1.

**Scheme 1:**
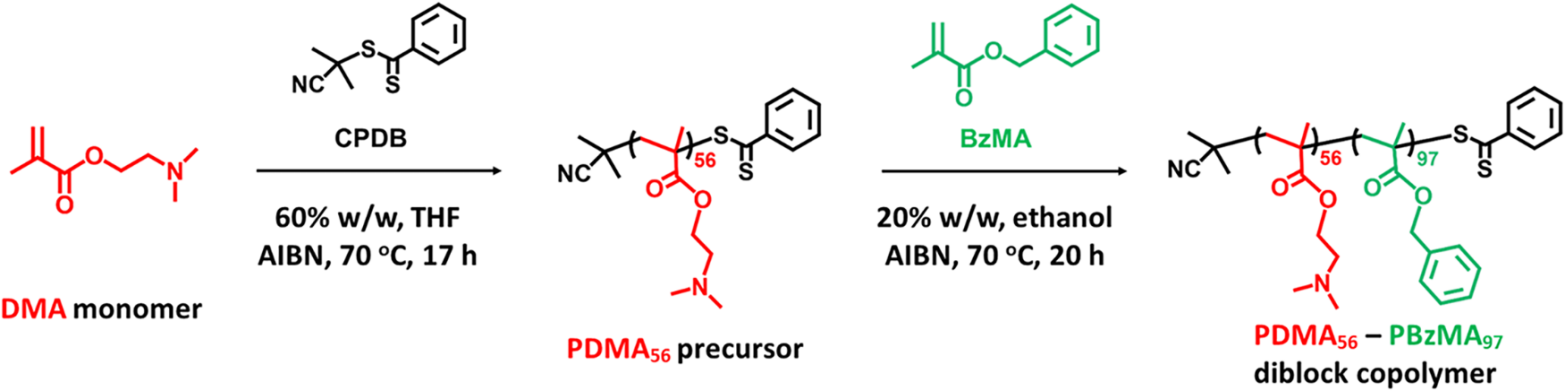
Synthesis of Poly­(2-(dimethylamino)­ethyl methacrylate) (PDMA_56_) via RAFT Solution Polymerization of DMA in THF at 60% w/w Solids Using 2-Cyano-2-propyl Dithiobenzoate (CPDB) RAFT Agent and 2,2′-Azobis­(isobutyronitrile) (AIBN) Initiator at 70 °C^a^

a This precursor was then chain-extended by RAFT alcoholic dispersion polymerization of benzyl methacrylate (BzMA) targeting 20% w/w solids at 70 °C in ethanol.

### Microcapsule Formulation

Microcapsules were readily prepared using the amphiphilic PDMA_56_-PBzMA_97_ stabilizer dissolved in the dispersed organic phase, in contrast to the conventional method where a water-soluble stabilizer (e.g., PVA) is dissolved in the continuous phase.^9^ This enabled an almost 100-fold reduction in the amount of required stabilizer for a typical formulation. During emulsification and subsequent microcapsule formation via solvent evaporation, it is reasonable to assume that the PDMA_56_-PBzMA_97_ chains were located at the DCM-water interface. The hydrophilic PDMA block should extend into the aqueous phase (and become protonated in the presence of acid), while the hydrophobic PBzMA block is expected to be well-solvated in the DCM phase, thus reducing the interfacial tension and conferring (electro)­steric stabilization to the newly formed interface. Accordingly, the pH of the continuous aqueous phase was lowered to pH 3 to ensure a high degree of protonation of the PDMA chains (p*K*_a_ ∼ 7.0–7.5).^17^

As the DCM slowly evaporates and the microcapsule shells are formed, the PDMA_56_-PBzMA_97_ stabilizer becomes anchored at the microcapsule-water interface through entanglements between the PBzMA block and the PBzMA homopolymer chains that form the microcapsule shell.^13^ This significantly enhances the colloidal stability of the microcapsules.^23^ Given the very low aqueous solubility of synthesized PDMA–PBzMA, any stabilizer that was not placed at the microcapsule-water interface likely partitioned into the bulk of the microcapsule shell matrix. Figure 1a shows perylene-loaded monolithic PBzMA microcapsules prepared by using the PDMA_56_-PBzMA_97_ stabilizer, which closely resemble PVA-stabilized microcapsules (Figure 1b) in terms of both morphology and particle size distribution (Figure S3). This is in striking contrast to the unsuccessful attempt to prepare poly­(lactic acid)-based microcapsules using a water-insoluble poly­(ethylene glycol)-poly­(lactic acid) copolymer (Figure S4). It is hypothesized that the PDMA_56_-PBzMA_97_ stabilizer confers superior stability via electrosteric stabilization, which is not possible for the nonionic poly­(ethylene glycol)-poly­(lactic acid) stabilizer. Andersson Trojer et al. previously prepared microcapsules using poly­(sodium methacrylate)-poly­(methyl methacrylate) (PMANa-PMMA) diblock copolymers.^23^ However, in this case, the best-performing stabilizer comprised a relatively long hydrophilic PMANa block and hence was preferentially located in the aqueous continuous phase, rather than the droplet phase. As discussed above, using a PDMA_56_-PBzMA_97_ stabilizer located within the oil droplets is much more efficient and does not affect the final microcapsule morphology.

**Figure 1:**
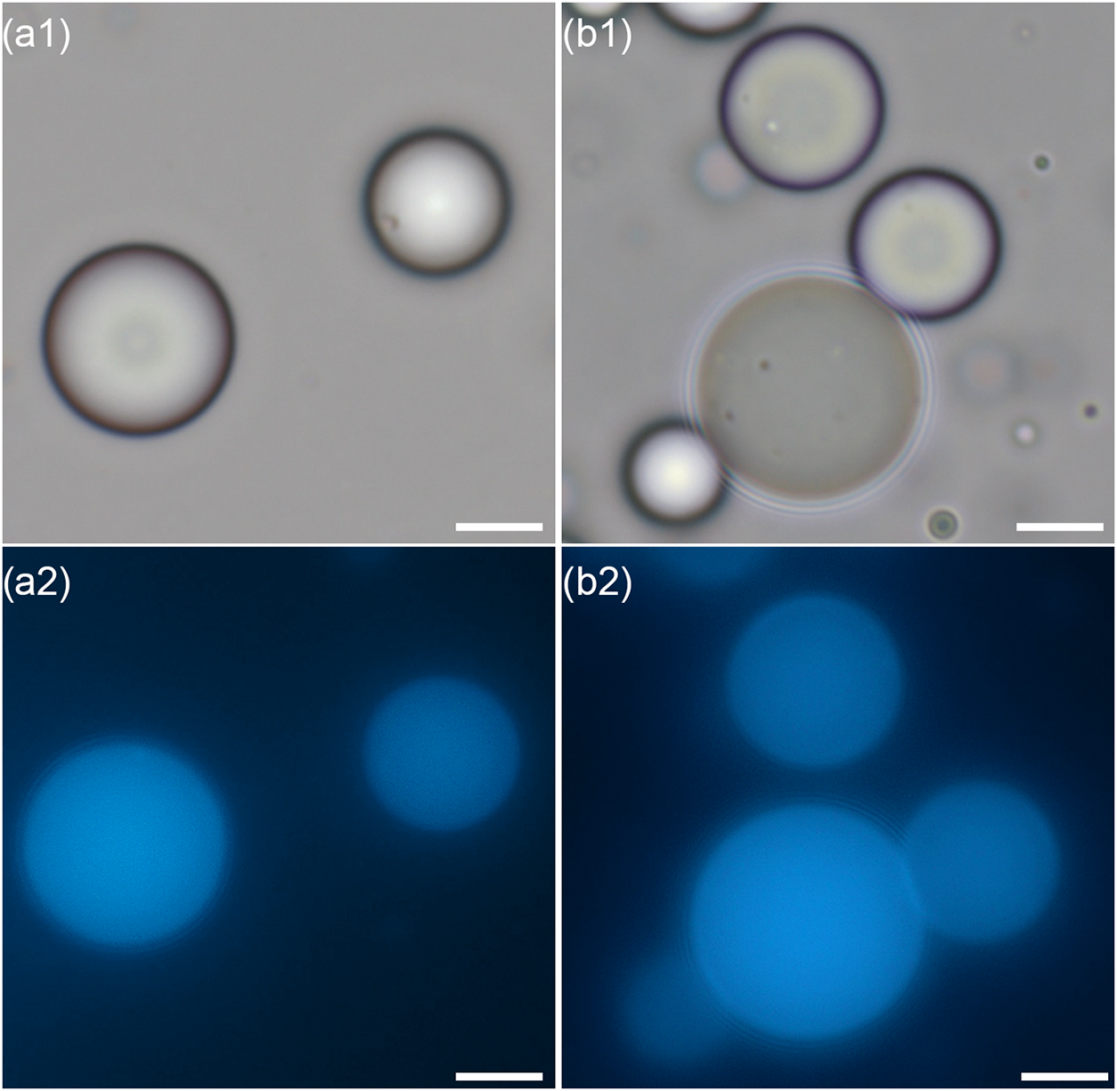
Perylene-loaded PBzMA monolithic microcapsules prepared using (a) PDMA_56_-PBzMA_97_ and (b) PVA. Images were acquired in the same field of view using (1) brightfield and (2) fluorescence microscopy (λ_ex_ = 365 nm, λ_em_ = 445 nm). All scale bars are 5 μm.

Aqueous electrophoresis data obtained for the PDMA_56_-PBzMA_97_-stabilized microcapsules are shown in Figure 2. As expected, positive ζ-potentials (at least +40 mV) were observed between pH 4.7 and pH 8.2 with an isoelectric point around pH 9. This is because the weakly basic PDMA chains exhibit a p*K*_a_ of around 7.0–7.5, so they are approximately 50% protonated at this pH.^17^ A very similar ζ-potential vs pH curve was observed for reconstituted PDMA_56_-PBzMA_97_ nanoparticles (and similar aqueous electrophoresis data were reported for comparable PISA-synthesized nanoparticles^17,18^ ). This confirms that the amphiphilic PDMA_56_-PBzMA_97_ chains are indeed located on the outer surface of the microcapsules.

**Figure 2:**
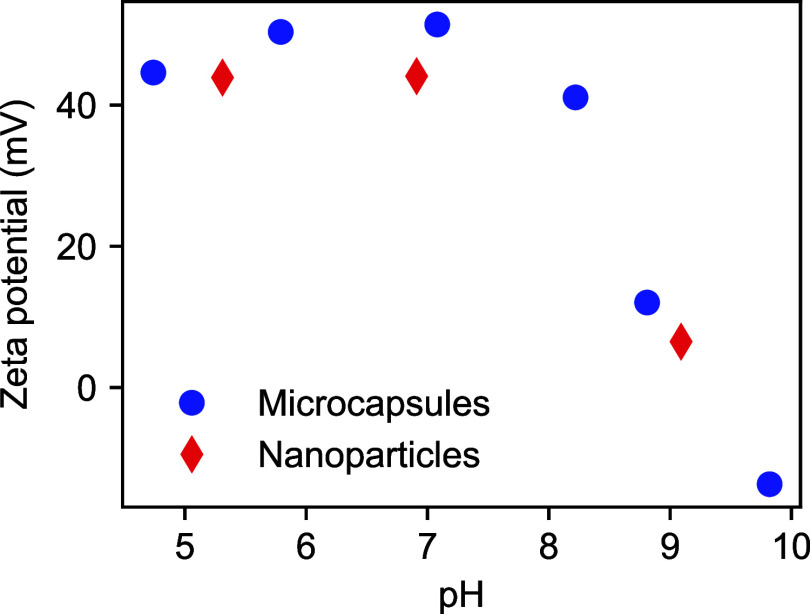
ζ-Potential vs pH curves constructed for the as-prepared PDMA_56_-PBzMA_97_-stabilized PBzMA microcapsules (blue data points) and an aqueous dispersion of PDMA_56_-PBzMA_97_ nanoparticles (red data points).

### Controlled Release of Encapsulated Actives

The fractional release of encapsulated pyrene from PBzMA microcapsules stabilized by either PDMA–PBzMA or PVA is shown in Figure 3a. Inspecting these measurements and the fitted diffusion coefficients in Figure 3b, it is clear that the microcapsule matrix determines the release rate rather than the type of stabilizer present on the microcapsule surface. This was verified by measuring the release from microcapsules stabilized by PDMA_56_-PBzMA_97_ when the PDMA block was in either its fully protonated (pH 3) or fully deprotonated (pH 9) form. Notably, the rate of release was around 3 orders of magnitude lower than that observed for PVA-stabilized poly­(d,l-lactide-*co*-glycolide) monolithic microcapsules^21^ and around 1 order of magnitude lower than PMMA monolithic microcapsules containing encapsulated pyrene (Figure S5). PBzMA is a highly hydrophobic polymer with a glass transition temperature, *T*_g_, of 54 °C (i.e., higher than the temperature at which the release of encapsulated pyrene was determined). Thus, the rate of release of pyrene from PBzMA was expected to be slower than that from water-plasticized PLGA microcapsules, for which the *T*_g_ is only around 30 °C.^24^ The *T*_g_ of the slightly less hydrophobic PMMA is 122 °C so the diffusivity of pyrene in this material was expected to be comparable to that for PBzMA. However, it is worth mentioning that PBzMA contains benzyl side groups rather than the methyl side groups of PMMA, which may influence the diffusivity of actives containing aromatic groups.

**Figure 3:**
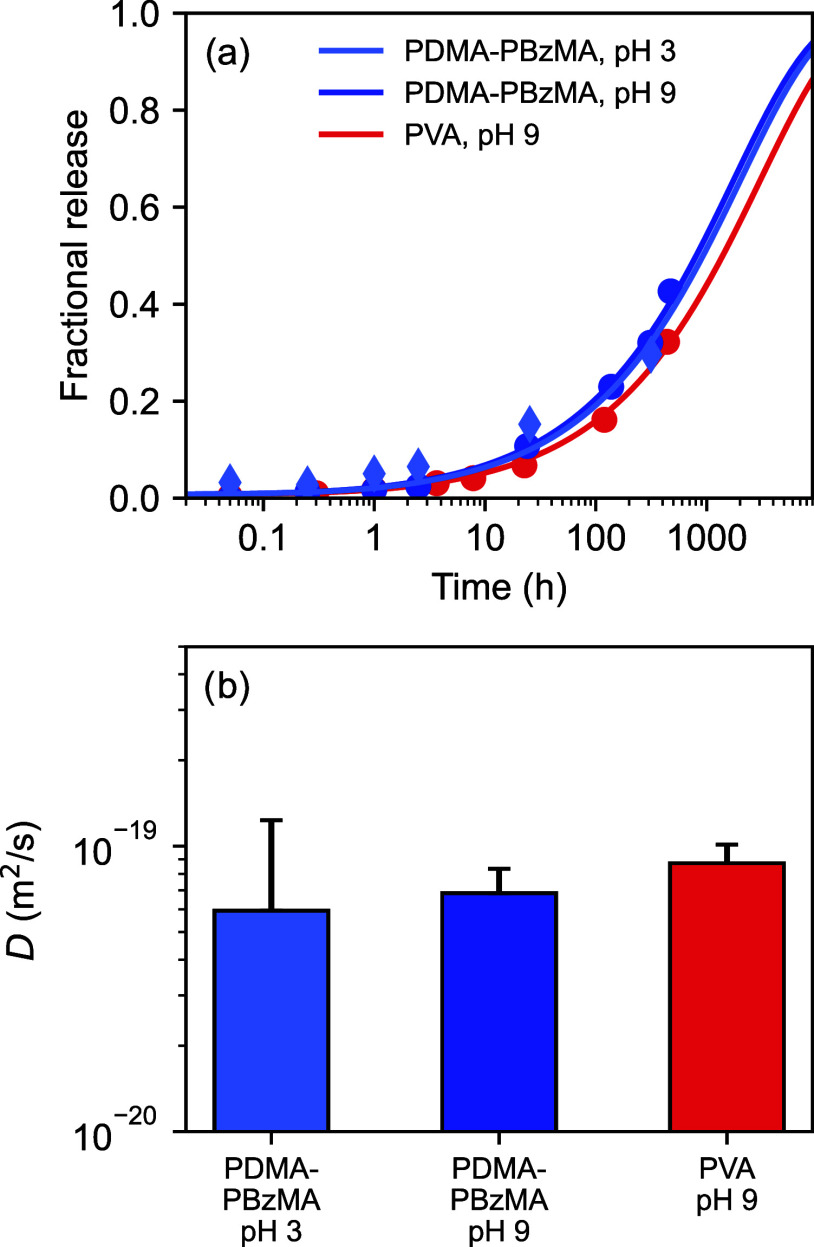
(a) Fractional release from PBzMA monolithic microcapsules containing 1% w/w encapsulated pyrene, stabilized using either PDMA_56_-PBzMA_97_ or PVA, respectively. The experimentally determined data points are shown together with fits based on a Fickian diffusion model. (b) Fitted diffusion coefficients derived from the release model.

### Versatility of PDMA_56_-PBzMA_97_ as a Microcapsule Stabilizer

To explore the versatility of PDMA_56_-PBzMA_97_ as a microcapsule stabilizer, two fundamentally different formulations were prepared. First, the shell material was altered to evaluate the compatibility of this amphiphilic diblock copolymer stabilizer with polymers other than PBzMA. Second, more complex formulations were evaluated, including oil-core microcapsules produced via internal phase separation and aqueous-core microcapsules created through *w*_1_/*o*/*w*_2_ double emulsification.

#### Oil Core Microcapsules

Figure 4 illustrates hexadecane-PMMA core–shell microcapsules prepared via internal phase separation using a PDMA_56_-PBzMA_97_ stabilizer. This combination of shell and core material has previously been extensively evaluated^8,13,23^ using a wide range of polymeric stabilizers. Unlike the PBzMA monolithic microcapsules formulated above, the formation of a core–shell morphology depends on the precise interfacial tensions between the shell, the core, and the aqueous phase.^8,9^ Like previous microcapsule formulations involving poly­(sodium methacrylate)-poly­(methyl methacrylate) stabilizers,^23^ it is assumed that the amphiphilic PDMA_56_-PBzMA_97_ stabilizer will significantly lower the shell-water interfacial tension during microcapsule formation. This should favor the formation of the desired core–shell morphology, both during intermediate stages and for the final microcapsules.^8^ Achieving and maintaining a core–shell morphology over a significant portion of the formation process is important to minimize core displacement toward one side of the microcapsule, which is critical for ensuring long-term mechanical stability.

**Figure 4:**
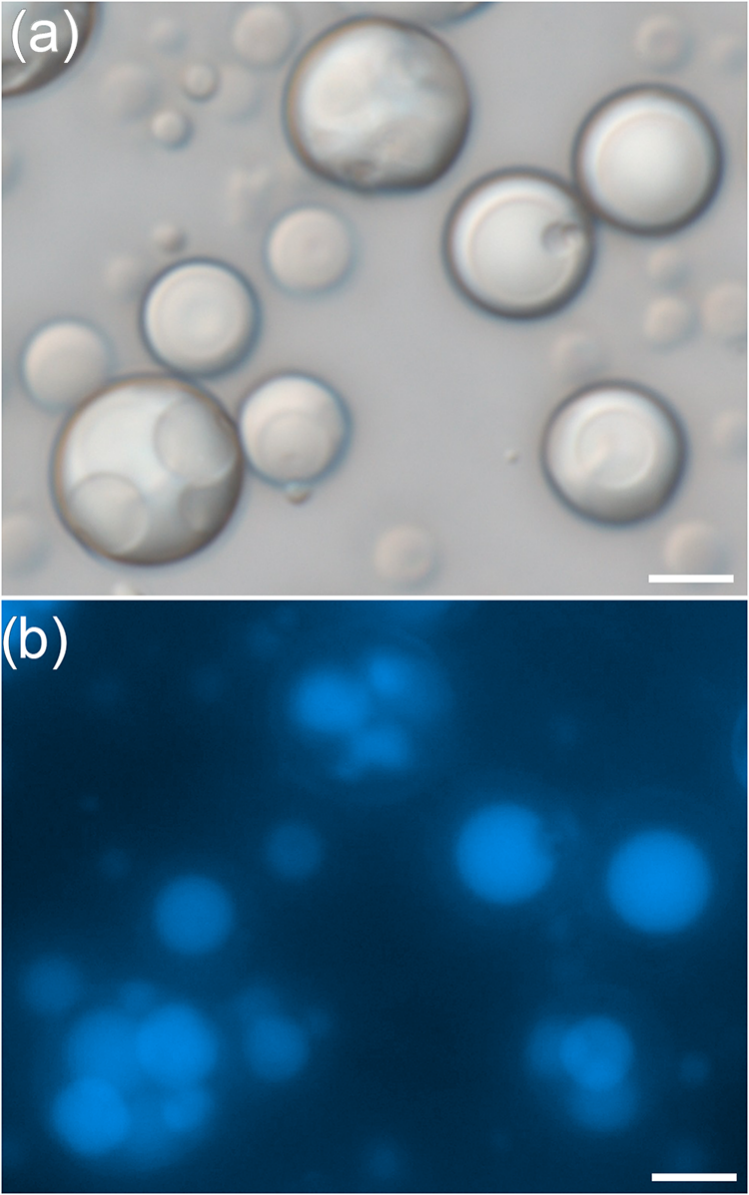
PMMA microcapsules with hexadecane cores prepared by using the PDMA_56_-PBzMA_97_ stabilizer. Images were acquired within the same field of view using (a) brightfield and (b) fluorescence microscopy. The scale bar is 5 μm.

Figures 4 and S6 confirm that the microcapsules exhibit a well-defined core–shell morphology. Most particles contain a single central core, with relatively few particles exhibiting multiple cores. This difference in the number of oil cores has been previously shown to be kinetically controlled during microcapsule formation.^8^ Fluorescence microscopy studies confirmed that a highly hydrophobic model compound, TIPS-An, predominantly partitioned into the core (Figure 4b). Moreover, all microcapsules exhibit fluorescence in their cores, confirming their structural integrity.

#### Aqueous-Core Microcapsules

Microcapsules comprising aqueous cores were prepared using a *w*_1_/*o*/*w*_2_ double emulsification method, see Figures 5 and S7. These microcapsules also displayed a core–shell morphology, albeit generated by a different mechanism. In contrast to the internal phase separation, where microcapsule formation is controlled by a combination of interfacial tensions and spreading coefficients, aqueous cores are kinetically stabilized by the amphiphilic PDMA_56_-PBzMA_97_ chains during an initial w/o emulsification step. After the second emulsification step, the oil phase was converted into shells via solvent evaporation.

**Figure 5:**
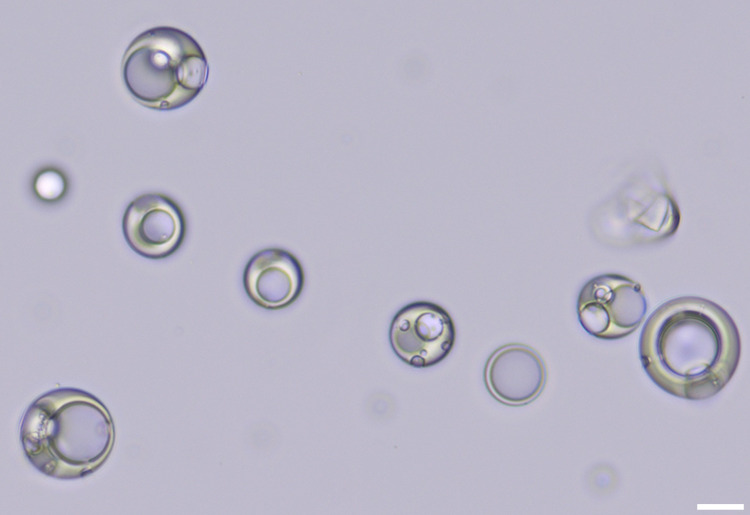
Aqueous-core PBzMA microcapsules were prepared using the PDMA_56_-PBzMA_97_ stabilizer. The scale bar is 10 μm.

In this case, the diblock copolymer stabilized both the inner and outer interfaces of the polymer shell. Achieving such dual-interface stabilization is usually challenging because polymeric (or surfactant) stabilizers are usually designed to preferentially dissolve in the continuous phase (which may be either oil or water).^25^ Consequently, conventional microcapsule formulations often require a judicious combination of oil-soluble and water-soluble stabilizers.^26^ Being soluble in the organic DCM phase of the *w*_1_/*o*/*w*_2_ emulsion, the PDMA_56_-PBzMA_97_ chains initially stabilized the precursor without emulsion via adsorption from the continuous phase. For the second emulsification step to form the final *w*_1_/*o*/*w*_2_ emulsion, the stabilizer was present in the dispersed organic phase. This dual functionality significantly simplifies the formulation process by eliminating the normal requirement to select appropriate pairs of water-soluble and oil-soluble stabilizers.

## Conclusions

An amphiphilic PDMA_56_-PBzMA_97_ diblock copolymer can be used for the preparation of monolithic microcapsules as well as both oil- and aqueous-core microcapsules. Unlike conventional water-soluble stabilizers, such as PVA, the PDMA_56_-PBzMA_97_ diblock copolymer was dissolved directly into the dispersed organic phase. This enabled stabilization of oil/water interfaces from within the dispersed organic phase, which minimizes its presence in the aqueous continuous phase. Moreover, dissolving PDMA–PBzMA in the organic phase enables the stabilizer concentration to be reduced by 2 orders of magnitude while eliminating additional processing steps to remove excess or unbound stabilizer from the continuous aqueous phase. Furthermore, this approach allows the stabilization of both the *w*_1_/*o* and *o*/*w*_2_ interfaces in a *w*_1_/*o*/*w*_2_ double emulsion, thus producing a double emulsion formulation using a single stabilizer. Importantly, PDMA–PBzMA-stabilized PBzMA monolithic microcapsules exhibit exceptionally slow release for encapsulated model actives, which highlights the release rate-limiting characteristics of the PBzMA microcapsule matrix. Using this new PDMA–PBzMA stabilizer also introduces functional benefits by conferring cationic character to the surface of the microcapsules. This surface functionality allows for precise control of microcapsule interactions, both attractive and repulsive, with anionic substrates. Strong anchoring of these functional groups potentially further enhances the long-term stability. Further functionalization through strongly anchored layer-by-layer buildup or electrostatic adhesion to surfaces for spatially confining the release of actives and providing long-term pH switchable attachment are easily conceived applications that broaden the scope of possible applications for the microcapsule systems presented in this work.

## Supplementary Material



## Notes

^1^H NMR spectrum and GPC curves of the diblock copolymer, microcapsule size distributions, photographs, micrographs, and release measurements of formulated microcapsules (PDF)

## References

[1] Bysell H., Månsson R., Hansson P., Malmsten M. (2011). Microgels and Microcapsules
in Peptide and Protein Drug Delivery. Adv. Drug
Delivery Rev..

[2] Lengyel M., Kállai-Szabó N., Antal V., Laki A. J., Antal I. (2019). Microparticles, Microspheres, and
Microcapsules for Advanced Drug
Delivery. Sci. Pharm..

[3] White A. L., Langton C., Wille M.-L., Hitchcock J., Cayre O. J., Biggs S., Blakey I., Whittaker A. K., Rose S., Puttick S. (2019). Ultrasound-Triggered
Release from
Metal Shell Microcapsules. J. Colloid Interface
Sci..

[4] Trojer M. A., Nordstierna L., Bergek J., Blanck H., Holmberg K., Nyden M. (2015). Use of Microcapsules as Controlled Release Devices for Coatings. Adv. Colloid Interface Sci..

[5] Rule J. D., Sottos N. R., White S. R. (2007). Effect of Microcapsule
Size on the
Performance of Self-Healing Polymers. Polymer.

[6] Augustin M. A., Hemar Y. (2009). Nano-and Micro-Structured Assemblies for Encapsulation of Food Ingredients. Chem. Soc. Rev..

[7] Esser-Kahn A. P., Odom S. A., Sottos N. R., White S. R., Moore J. S. (2011). Triggered
Release from Polymer Capsules. Macromolecules.

[8] Eriksson V., Edegran S., Croy M., Evenäs L., Andersson Trojer M. (2024). A Unified Thermodynamic and Kinetic
Approach for Prediction
of Microcapsule Morphologies. J. Colloid Interface
Sci..

[9] Loxley A., Vincent B. (1998). Preparation of Poly­(Methylmethacrylate) Microcapsules
with Liquid Cores. J. Colloid Interface Sci..

[10] Yan J., Mangolini F. (2023). Polymer-Encapsulated
Ionic Liquids as Lubricant Additives
in Non-Polar Oils. J. Mol. Liq..

[11] Yang Y., Chen Y., Hou Z., Li F., Xu M., Liu Y., Tian D., Zhang L., Xu J., Zhu J. (2020). Responsive
Photonic Crystal Microcapsules of Block Copolymers with Enhanced Monochromaticity. ACS Nano.

[12] Trojer M. A., Andersson H., Li Y., Borg J., Holmberg K., Nydén M., Nordstierna L. (2013). Charged Microcapsules for Controlled
Release of Hydrophobic Actives. Part III: The Effect of Polyelectrolyte
Brush- and Multilayers on Sustained Release. Phys. Chem. Chem. Phys..

[13] Trojer M. A., Holmberg K., Nydén M. (2012). The Importance
of Proper Anchoring
of an Amphiphilic Dispersant for Colloidal Stability. Langmuir.

[14] Chiefari J., Chong Y., Ercole F., Krstina J., Jeffery J., Le T. P., Mayadunne R. T., Meijs G. F., Moad C. L., Moad G. (1998). Living
Free-Radical Polymerization by Reversible Addition-Fragmentation
Chain Transfer: The RAFT Process. Macromolecules.

[15] Moad G., Rizzardo E., Thang S. H. (2005). Living
Radical Polymerization by
the RAFT Process. Aust. J. Chem..

[16] Perrier S. (2017). 50th Anniversary
Perspective: RAFT Polymerization—A User Guide. Macromolecules.

[17] Jones E. R., Semsarilar M., Blanazs A., Armes S. P. (2012). Efficient Synthesis
of Amine-Functional Diblock Copolymer Nanoparticles via RAFT Dispersion
Polymerization of Benzyl Methacrylate in Alcoholic Media. Macromolecules.

[18] Semsarilar M., Jones E. R., Blanazs A., Armes S. P. (2012). Efficient Synthesis
of Sterically-Stabilized Nano-Objects via RAFT Dispersion Polymerization
of Benzyl Methacrylate in Alcoholic Media. Adv.
Mater..

[19] Trojer M. A., Gabul-Zada A. A., Ananievskaia A., Nordstierna L., Östman M., Blanck H. (2019). Use of Anchoring Amphiphilic Diblock
Copolymers for Encapsulation of Hydrophilic Actives in Polymeric Microcapsules:
Methodology and Encapsulation Efficiency. Colloid
Polym. Sci..

[20] Eriksson V., Nygren E., Bordes R., Evenäs L., Trojer M. A. (2024). Electrostatically Hindered Diffusion for Predictable
Release of Encapsulated Cationic Antimicrobials. RSC Pharm..

[21] Eriksson V., Mistral J., Nilsson T. Y., Trojer M. A., Evenäs L. (2023). Microcapsule
Functionalization Enables Rate-Determining Release from Cellulose
Nonwovens for Long-Term Performance. J. Mater.
Chem. B.

[22] Crank, J. The Mathematics of Diffusion; Oxford University Press, 1979.

[23] Trojer M. A., Li Y., Abrahamsson C., Mohamed A., Eastoe J., Holmberg K., Nydén M. (2013). Charged Microcapsules
for Controlled Release of Hydrophobic
Actives. Part I: Encapsulation Methodology and Interfacial Properties. Soft Matter.

[24] Blasi P., D’Souza S. S., Selmin F., DeLuca P. P. (2005). Plasticizing Effect
of Water on Poly­(Lactide-Co-Glycolide). J. Controlled
Release.

[25] Evans, D. F. ; Wennerström, H. The Colloidal Domain: Where Physics, Chemistry, Biology, and Technology Meet, 2nd ed.; Wiley-VCH, 1999.

[26] Garti N. (1997). Double Emulsions
— Scope, Limitations and New Achievements. Colloids Surf., A.

