# Supplementary material for: Preparation of Microcapsules Using a Poly(2-(dimethylamino)ethyl methacrylate)‑b‑poly(benzyl methacrylate) Diblock Copolymer Emulsifier
Source: Langmuir. 2025 Dec 24;42(1):392–9. doi: 10.1021/acs.langmuir.5c04262 (PMC12810376; doi:10.1021/acs.langmuir.5c04262)
Supplement: Supplementary file 1 [file la5c04262_si_001.pdf]

Preparation of microcapsules using a poly(2-(dimethylamino)ethyl methacrylate)-b-poly(benzyl methacrylate) diblock copolymer emulsifier

Viktor Kallebäck[1,2,*], Csilla György[3], Gustav Eriksson[2], Steven P. Armes[3], Markus L. Andersson Trojer[1,2], Lars Evenäs[2]

[1]Department of Sustainable Material Systems, RISE Research Institutes of Sweden, 431 53 Mölndal, Sweden

[2]Department of Chemistry and Chemical Engineering Chalmers University of Technology, 412 96 Gothenburg, Sweden

[3]Dainton Building, School of Mathematical and Physical Sciences, University of Sheffield, Sheffield, Brook Hill, Sheffield, South Yorkshire, S3 7HF, UK

[*]Corresponding author

PDMA-PBzMA Synthesis and Characterization

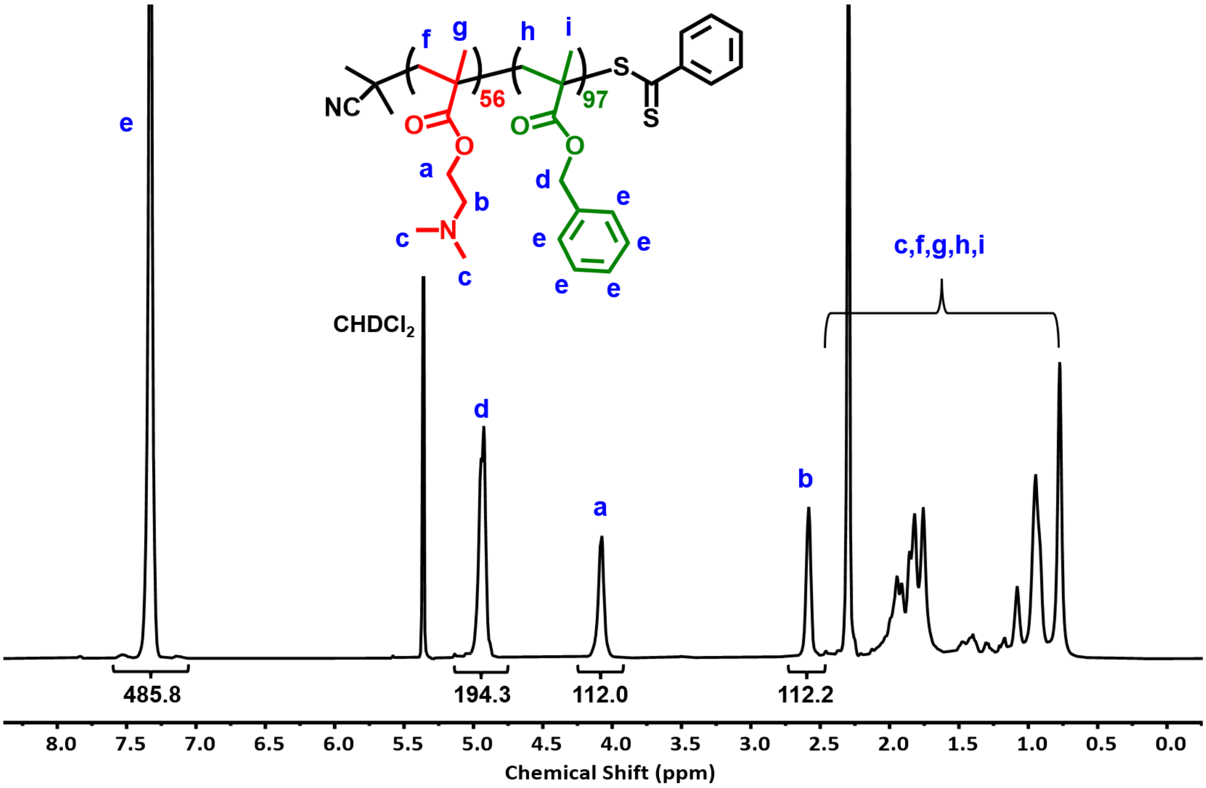

Figure S1. Assigned integrated ^{1}H-NMR spectrum recorded in CD_2Cl_2 for the $PDMA_{56}$-$PBzMA_{97}$ diblock copolymer emulsifier used in this study.

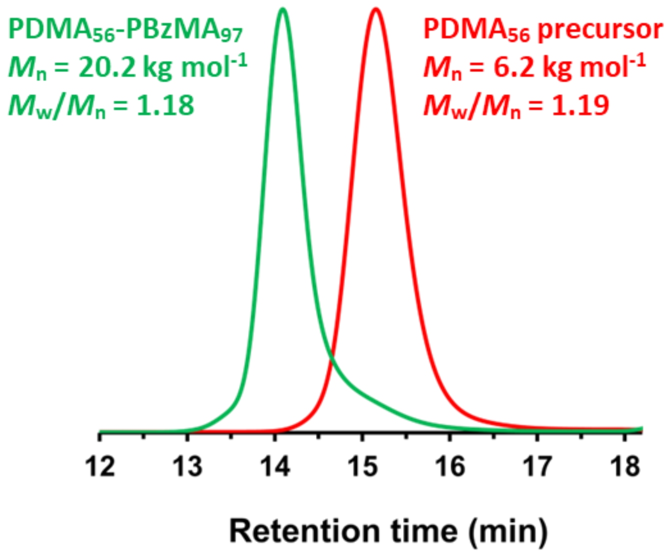

Figure S2. Chloroform GPC curves (refractive index detector; *vs.* a series of near-monodisperse poly(methyl methacrylate) calibration standards) recorded for the $PDMA_{56}$ precursor and the $PDMA_{56}$-$PBzMA_{97}$ diblock copolymer.

Microcapsule characterization

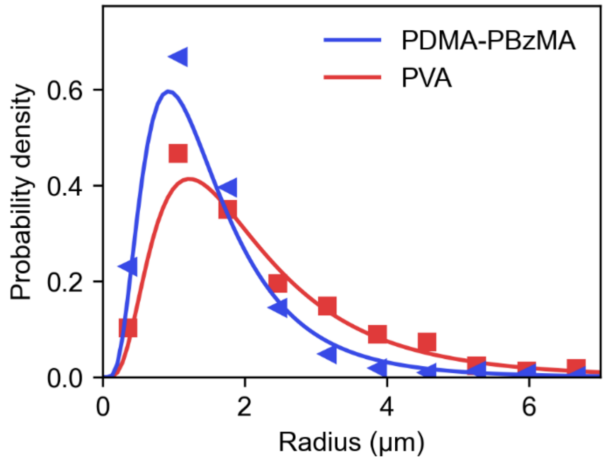

Figure S3. Size distributions from optical microscopy obtained for PBzMA microcapsules stabilized using either $PDMA_{56}$-$PBzMA_{97}$ or PVA, respectively.

Attempted formulation of microcapsules using a poly(ethylene glycol)-poly(lactic acid) stabilizer

A formulation of poly(L-lactic acid) (PLLA, 40-70 kg mol^{-1}, Polysciences) containing ethyl linoleate (99%, Sigma-Aldrich) was prepared by addition of the water-insoluble diblock copolymer poly(ethylene glycol)-poly(lactic acid) (PEG-PLA, 5000 g mol^{-1}-1000 g mol^{-1}, Polysciences) to the organic phase (DCM) at concentrations between 2% and 16% w/w of the microcapsule shell matrix – far higher than the amounts of $PDMA_{56}$-$PBzMA_{97}$ used in the main article. Methylene blue (96%, Riedel-de Haën) was added as a fluorescent dye. **Figure S4** shows the aqueous and DCM phase stabilized by PEG-PLA during homogenization. Clearly, it

is not possible to produce a stable DCM-in-water emulsion and consequently PLLA microcapsules could not be formulated using PEG-PLA as a stabilizer.

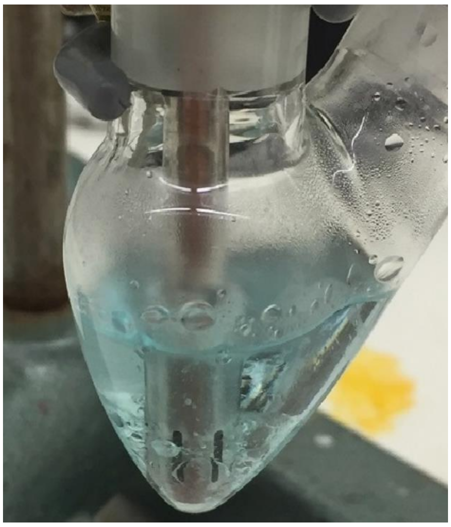

Figure S4. Photograph captured during the attempted formulation of polylactide microcapsules using a poly(ethylene glycol)-poly(lactic acid) diblock copolymer stabilizer.

Controlled release from PMMA microcapsules

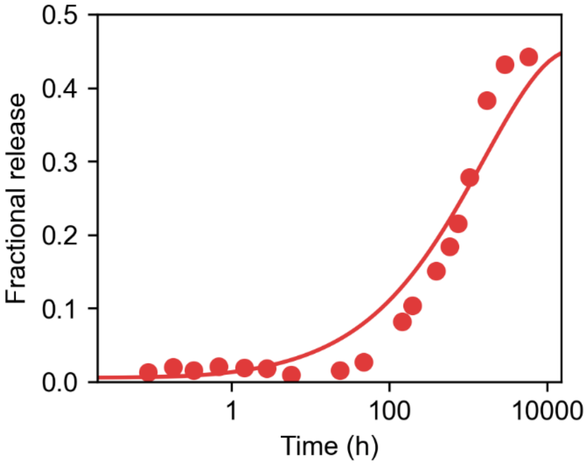

Figure S5. Fractional release of pyrene from PVA-stabilized monolithic PMMA microcapsules. The experimentally determined data points are shown together with fits based on a Fickian diffusion model.

Core-shell microcapsule formulation

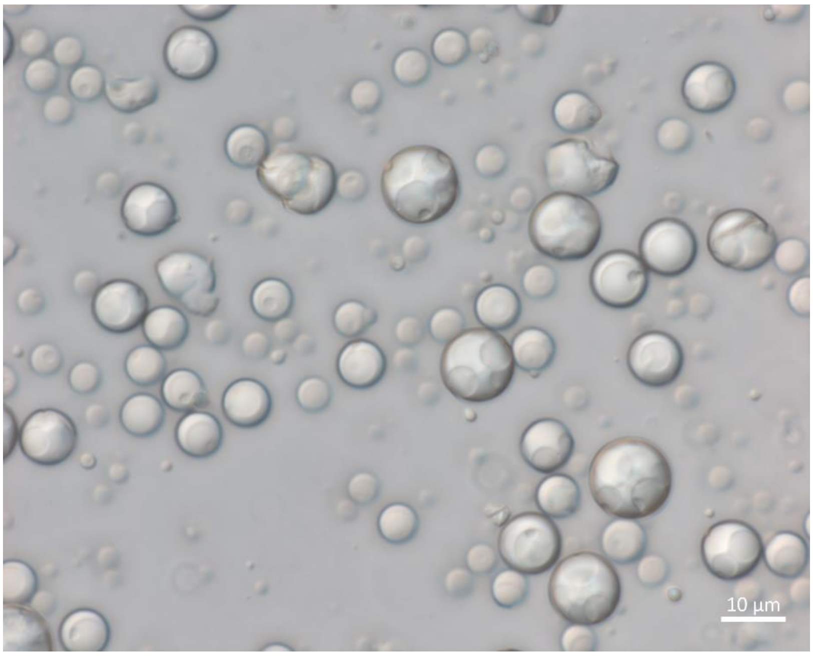

Figure S6. PMMA microcapsules with hexadecane cores prepared using the PDMA$_{56}$-PBzMA$_{97}$ stabilizer.

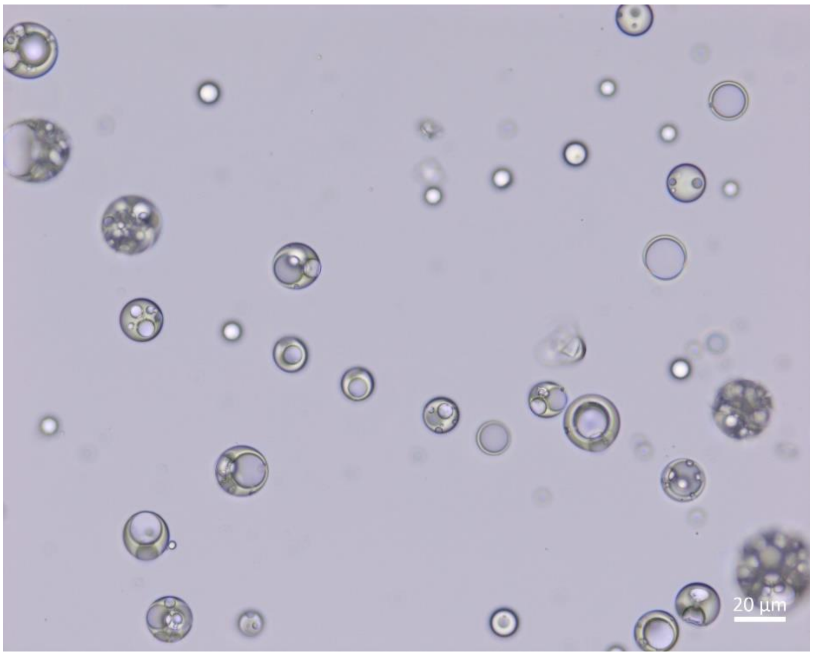

Figure S7. Aqueous-core PBzMA microcapsules prepared using the PDMA$_{56}$-PBzMA$_{97}$ stabilizer.
